# Lost in Navigation: A Simplified CT-Based Airway Mapping Method for Ultrathin Navigational Bronchoscopy

**DOI:** 10.3390/jcm14228009

**Published:** 2025-11-12

**Authors:** Nathan Mortimer, Tajalli Saghaie

**Affiliations:** Macquarie University Hospital, Sydney 2109, Australia; tajalli.saghaie@mqhealth.org.au

**Keywords:** navigation, bronchoscopy, bronchial branch tracing, peripheral pulmonary lesion, navigational bronchoscopy

## Abstract

Accurate navigation within the bronchial tree is essential for successful bronchoscopic sampling of peripheral pulmonary lesions. It fundamentally comprises a series of di/trichotomous decisions as the bronchoscope is advanced through sequential airway generations. This letter describes a simplified, pen-and-paper method for bronchial branch tracing that minimizes distracting complexity and streamlines airway mapping into reproducible steps that can be rapidly performed with reference to pre-procedural CT images. By anchoring navigation in anatomical recognition and spatial awareness, this technique offers both a reliable primary strategy and a dependable fallback option for bronchoscopists seeking clarity and confidence in the procedural suite.

## 1. Introduction

Successful airway navigation is fundamental to the safe and accurate bronchoscopic sampling of peripheral pulmonary lesions. As the field of interventional pulmonology continues to expand, there is a growing need for bronchoscopists who can confidently negotiate multiple generations of sub-segmental airway anatomy.

Recent evidence supports the combination of ultrathin bronchoscopy with manual bronchial branch tracing (BBT) for navigation towards peripheral pulmonary lesions. In a 2024 single-centre retrospective study of 47 patients undergoing ultrathin bronchoscopy with radial EBUS, navigational success with manual BBT was 91.5% [1]. Earlier published series and reviews on the use of ultrathin bronchoscopy for evaluation of peripheral lesions indicate variable efficacy and diagnostic yields ranging from 55 to 82%, although any manual navigational methods applied in such series are infrequently described in detail [2]. A large cross-sectional study evaluating the yield of bronchoscopic brushing before and after implementation of manual BBT in clinical practice identified significant improvements in yield, although without the use of ultrathin bronchoscopes [3]. Several studies have compared manual path planning methods with virtual bronchoscopic navigation and found comparable diagnostic yields and the ability to identify a greater number of airway bifurcations, particularly in the lung periphery [4,5,6]. Manual BBT has also been reported as an effective fallback strategy in cases where errors of divergence have hindered electromagnetic navigation with a robotic platform [7]. However, it remains challenging to draw any firm conclusions regarding the impact of BBT on yield in bronchoscopic procedures (which is compounded by the variable applied definitions) as it represents a composite endpoint of multiple procedural, patient, and cohort factors. Future comparative or cohort studies on the topic would be better served by evaluating navigational performance as a primary outcome.

Technologies designed to support airway navigation are often costly, time-consuming, and may have limited global availability. Navigation systems that rely on tracking pulmonary anatomy and lesions in three-dimensional space while correlating with a pre-procedural CT scan are inherently prone to errors of CT-to-body divergence [8]. When they fail, the bronchoscopist may be left without a fallback option. Manual BBT is not susceptible to CT-to-body divergence as it simply relies on the sequential recognition of endobronchial airway anatomy.

The Kurimoto method of BBT is the most established technique for manually preparing an airway map suitable for navigation [9]. This detailed approach comprises strictly defined steps that are effective once mastered, although challenging to learn and teach. It is important to remember that the purpose of any navigation strategy is to assist the bronchoscopist in choosing the correct airway at each bifurcation in order to reach their intended destination in the bronchial tree. For each procedure, the bronchoscopist is likely to face no more than four or five such choices. Therefore, a practical navigation strategy does not require perfect fidelity or a life-like appearance to achieve its purpose. In fact, unnecessary attention to detail could potentially become distracting and add to the complexity of the process.

The simplified approach to BBT described below is designed to be intuitive, versatile, and can be performed rapidly with a high degree of accuracy once sufficiently practiced. It also supports the bronchoscopist in maintaining a detailed understanding of airway anatomy, which in turn enhances spatial awareness of the bronchial tree and its relationship with the target lesion for an optimal biopsy approach.

## 2. Simplified Bronchial Branch Tracing—Step by Step

**Figure 1:**
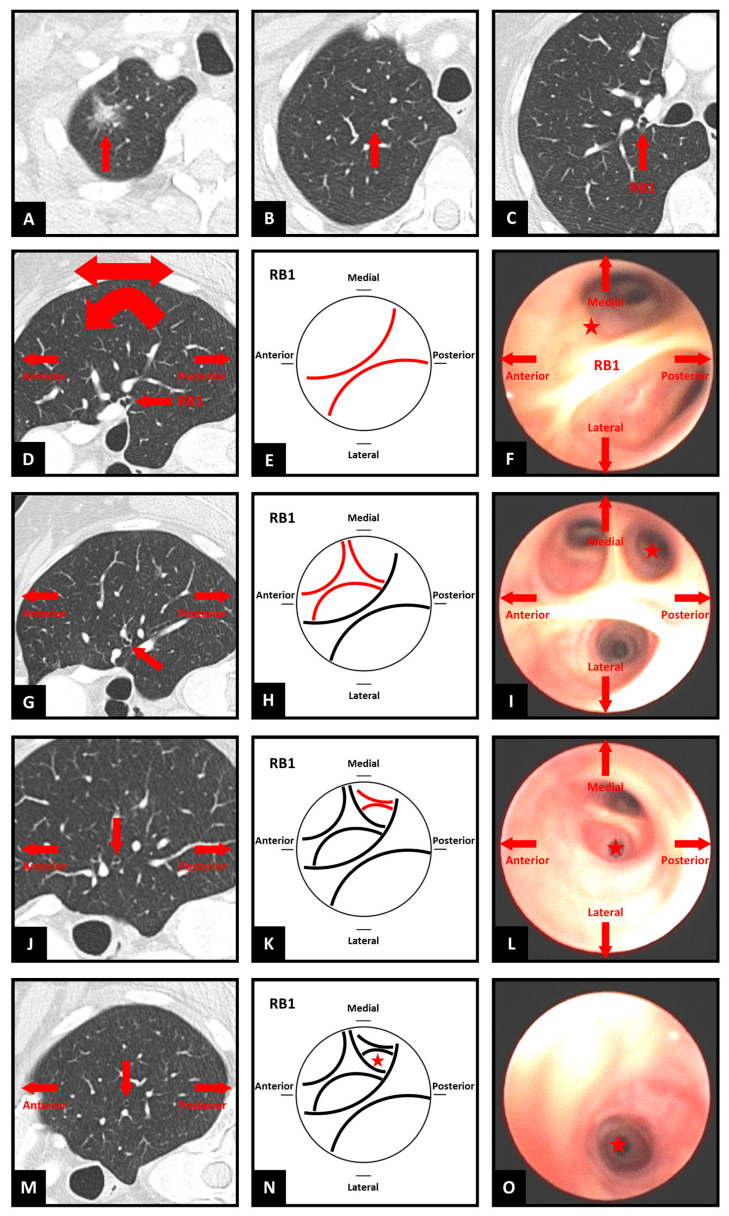
Steps of simplified bronchial branch tracing. RB1 = right upper lobe apical segment bronchus. ★ symbol indicates target airway for navigation. (**A**–**C**) Identifying the segmental airway; (**D**) CT image transformation; (**E**) Drawing the segmental ostium; (**F**) Starting position and orientation; (**G**) Finding the next generation; (**H**,**I**) Drawing the branches; (**J**–**L**) Mapping the generations; (**M**–**O**) Marking the target airway.

**Step 1. Choose the destination and identify the segmental airway (Figure 1A–C)**

To identify which segmental airway leads to the target, first identify the sub-segmental airway that leads to the target (if a bronchus sign is present) or is most favorable for accessing the target (if no bronchus sign is present). Then, move through the CT slices to progress proximally through the preceding generations of airways to arrive at the ostium of the segmental airway.

**Step 2. Transform the CT image—optional (Figure 1D)**

**Figure 2:**
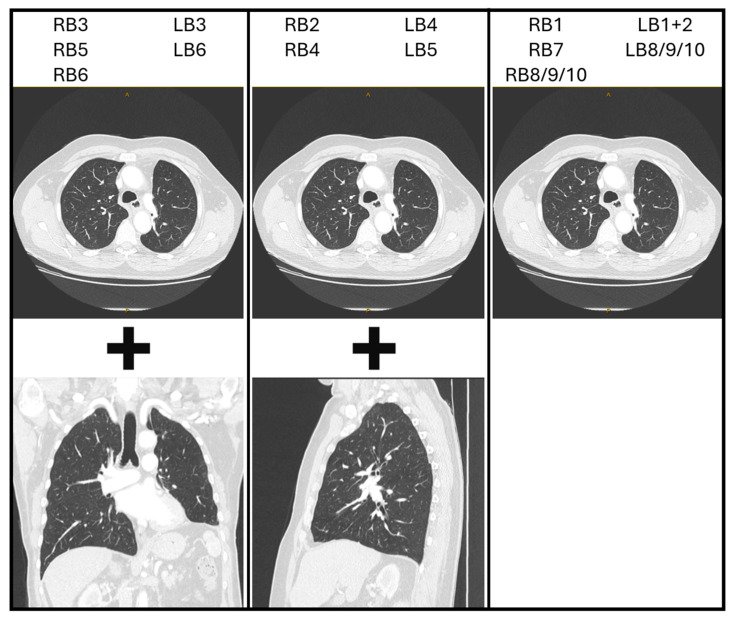
Potential CT image reconstructions for each segmental bronchus. RB = right segmental bronchi. LB = left segmental bronchi. For example, if navigating via the RB3 segmental airway, consider using axial images (with a horizontal flip ± 90-degree counterclockwise rotation) and coronal reconstructions.

Transformation (flipping and/or rotation) of the CT image is not essential, provided the bronchoscopist can maintain a spatial awareness of the anatomical directions as they draw the map. These anatomical directions are often marked on the edges of the CT images as well. Each bronchoscopist can therefore decide if they prefer to perform such transformations on the CT image or in their mind. However, it may be more instinctive if axial images are transformed in a manner that assists the bronchoscopist in imagining themselves navigating the CT images as if they were performing the bronchoscopy. Therefore, the authors suggest initially flipping the image horizontally to reflect the anatomical orientation as the bronchoscope enters the trachea, then rotating 90 degrees clockwise or counterclockwise for left and right-sided lesions, respectively. This reflects the rotation of the bronchoscope as occurs when the left or right main bronchus is intubated. Subsequent transformations may be natural for some users, such as flipping the CT image vertically for upper lobe lesions in the apical segments. It may also be helpful to use sagittal reconstructions for laterally projecting segmental airways and coronal reconstructions for anteriorly/posteriorly projecting segmental airways. Because the ostium of a segmental airway is rarely facing in a precise anatomical direction, consider either using the closest direction or a combination of axial and reconstructed CT images side-by-side. Potential combinations are suggested in Figure 2, although considering the individual anatomy on a case-by-case basis is most reliable.

**Step 3. Determine the starting position and orientation (Figure 1F)**

Imagine yourself positioned as the bronchoscope at the ostium of the segmental airway that leads to the target. Using the CT images, establish your orientation relative to the patient as you directly face to enter the segmental airway. What part of the patient is above and below you, and to your left and right? Match these four directions with their closest corresponding anatomical directions (superior, inferior, medial, lateral, anterior, posterior). Existing familiarity with flexible bronchoscopy is essential to understanding how the bronchoscope will be positioned. As segmental airways rarely align with precise anatomical directions, consider either using the closest direction or a combination, for example, antero-lateral, postero-medial, etc.

**Step 4. Draw the segmental ostium (Figure 1E)**

Draw a circle, which represents the bronchoscopic view at the ostium of the segmental airway, then indicate the four anatomical directions around the circle. This forms the basis for the map.

**Step 5. Find the next generation (Figure 1G)**

Starting at the segmental ostium, move through the CT slices and imagine you are proceeding along the airway until you identify the next carina/generation. There will be either two or three branches. By moving back and forth, determine the relative position and anatomical direction of these two or three branches. For example, one branch may be projecting more medially in relation to another branch projecting more laterally

**Step 6. Draw the branches (Figure 1H,I)**

Inside the circle, draw an arc corresponding to each branch in the approximate position relative to the other branch(es) and direction relative to the four anatomical directions around the circle. Try to approximately reflect the relative sizes of the branches on the map. Remember that the map does not need to be perfect. So long as the relative anatomical direction of each airway is accurately reflected, the map will achieve its purpose of guiding the choice between the branches at each carina/generation.

**Step 7. Map subsequent generations to the target airway (Figure 1J–L)**

Repeat the process (from step 5) for each successive generation of airway leading to the target or destination airway, drawing arcs within the respective preceding arcs.

**Step 8. Mark the target airway on the map (Figure 1M–O)**

If there are multiple sub-segmental airways that lead to the target, you can indicate multiple options on the map.

## 3. Comparison of Original BBT and the Simplified Approach

The changes in the simplified approach aim to minimise cognitive load and condense the mapping and navigation process to its most fundamental purpose-deciding the path to take at each bifurcation.

**Step 2:** Whereas the original BBT method defines the CT image transformations for each lobe or segmental airway, the simplified approach advises an individualised approach, which allows for anatomical variability and proceduralist preference.

**Step 3:** The original method emphasises individual segmental directionality, positioning of the patient’s head, and a segmental airway viewpoint. The simplified approach condenses this to a single concept wherein the proceduralist imagines the view from the bronchoscope at the segmental airway, then determines the orientation relative to the patient with the help of the CT images.

**Step 4:** The simplified approach emphasises the anatomical directions with reference to the starting position as the basis for subsequent mapping, compared with the original method, in which mapping is more prescriptive, based on numerous anatomical possibilities.

**Step 5:** In contrast to the four patterns (vertical, horizontal–horizontal, horizontal–vertical, and horizontal–oblique) of branch tracing described in the original method, the simplified approach primarily considers the relative direction of each branch in reference to the mapped anatomical directions in the previous step.

**Steps 6–8:** The simplified approach adopts a process of drawing nested arcs to represent the airways and dispenses with complex branch naming conventions in favour of simple annotation when mapping for the purpose of navigation.

## 4. Principles and Tips

**CT Image Transformation**

Each bronchoscopist may choose to transform the CT image in a way that aligns with their own spatial understanding and the way they intubate certain segments. Although it is possible to memorise the most common CT image transformations for each segmental airway, it is the authors’ opinion that considering the transformations on a case-by-case basis improves spatial awareness and reduces error. This approach accommodates variability between bronchoscopists in how they choose to navigate/intubate the airways, as well as anatomical variants and pathologic distortion.

**Airway Identification**

It is highly recommended to use thin-slice CT images to avoid missing small sub-segmental airways when moving through the slices. A combination of axial and coronal/sagittal reconstructed CT images can assist in detecting small sub-segmental airways.

Looking for ‘breaks’ in the bronchial wall on the CT image can help identify where tiny sub-segmental airways may originate. Furthermore, identifying small pulmonary arteries can help find the accompanying small sub-segmental airways, as they generally run together. One can differentiate pulmonary arteries from bronchial arteries or pulmonary veins by tracing them proximally.

**Complex Mapping and Navigation**

When using the map to navigate, be aware that the orientation of the bronchoscopic image will change as the bronchoscope is rotated to enter subsequent generations of airways. The bronchoscopist can either correct the orientation of the bronchoscope relative to the patient after entering each sub-segment or change the orientation of the map (physically or mentally). Fluoroscopy can also be helpful to assist with this correction or adjusting orientation.

Numbering each carina/generation on the map in the order of their anticipated appearance can help visually navigate more complex maps. It may also be helpful to draw one or two successive airway generations within the branches that do not lead to the target. When using the map to navigate, this can serve as an additional point of reference and help recognise incorrect paths.

## 5. Conclusions

Just as bronchoscopic technique varies among operators, so too do the strategies for navigating the bronchial tree. The approach to bronchial branch tracing described here seeks to streamline existing manual methods, offering a straightforward yet dependable way of preparing airway maps. By grounding navigation in anatomy rather than image–registration technologies, it avoids the pitfalls of CT-to-body divergence and provides a reliable means of maintaining orientation when other systems falter. In this way, it can serve both as a practical primary strategy and as an essential safeguard when technology proves flustered. By reducing complexity without diminishing accuracy, this method has the potential to make airway mapping more accessible, reproducible, and enduring in clinical practice.

## Abbreviations

The following abbreviations are used in this manuscript:

CT	Computed Tomography
BBT	Bronchial Branch Tracing
